# Mucus Properties and Goblet Cell Quantification in Mouse, Rat and Human Ileal Peyer's Patches

**DOI:** 10.1371/journal.pone.0083688

**Published:** 2013-12-16

**Authors:** Anna Ermund, Jenny K. Gustafsson, Gunnar C. Hansson, Åsa V. Keita

**Affiliations:** 1 Department of Medical Biochemistry, University of Gothenburg, Gothenburg, Sweden; 2 Department of Clinical and Experimental Medicine, Faculty of Health Sciences, Linköping University and County Council of Östergötland, Linköping, Sweden; Indian Institute of Science, India

## Abstract

Peyer's patches (PPs) are collections of lymphoid follicles in the small intestine, responsible for scanning the intestinal content for foreign antigens such as soluble molecules, particulate matter as well as intact bacteria and viruses. The immune cells of the patch are separated from the intestinal lumen by a single layer of epithelial cells, the follicle-associated epithelium (FAE). This epithelium covers the dome of the follicle and contains enterocyte-like cells and M cells, which are particularly specialized in taking up antigens from the gut. However, the presence and number of goblet cells as well as the presence of mucus on top of the FAE is controversial. When mouse ileal PPs were mounted in a horizontal Ussing-type chamber, we could observe a continuous mucus layer at mounting and new, easily removable mucus was released from the villi on the patch upon stimulation. Confocal imaging using fluorescent beads revealed a penetrable mucus layer covering the domes. Furthermore, immunostaining of FAE from mice, rats and humans with a specific antibody against the main component of intestinal mucus, the MUC2 mucin, clearly identify mucin-containing goblet cells. Transmission electron micrographs further support the identification of mucus releasing goblet cells on the domes of PPs in these species.

## Introduction

While the gastrointestinal tract epithelium must be accessible for nutrient absorption, with ingested food comes foreign antigens, commensal microorganisms in addition to potentially harmful bacteria and viruses. The same concept applies to the airways, where gas exchange takes place while air borne pathogens and particulate matter as well as chemicals meet the airways. To protect against assault, the mucosal surfaces are covered in a protective mucus layer consisting of mainly secreted mucins, MUC5AC in the airways and stomach and MUC2 in the intestine.

The MUC2 mucin forms a two layered mucus system in the colon, where the inner layer is anchored to the epithelium and devoid of bacteria whereas the outer layer is freely movable and forms a habitat for the commensal flora [1]. In contrast, the small intestine has only one type of mucus, which is not anchored to the epithelium but still built around the MUC2 mucin [2], [3]. In the small intestine the Paneth cells and enterocytes secrete numerous antimicrobial peptides and proteins [4]. As the mucus acts as a diffusion barrier, these antibacterials are not quickly lost but form a gradient from the epithelium toward the lumen. Combined with the slow diffusion of bacteria in the mucus, this will limit contact between luminal bacteria and the epithelium [5]. Additionally, the mucosal immune system conducts immune surveillance of the luminal content, in order to provide protection against pathogens as well as tolerance to the commensal flora and other antigens [6]. Along the GI tract and airways, specialized structures called mucosal lymphoid follicles facilitate the interaction between epithelial cells and cells of the immune system [7]. There are both isolated and aggregated lymphoid follicles in the gut. The aggregated lymphoid follicles form the Peyer's patches (PPs). Lymphoid follicles consist of a B cell germinal center, a marginal zone, where B cells and macrophages reside, and the subepithelial dome, where T cells, B cells, macrophages and dendritic cells can be found. Several follicles separated by interfollicular regions, made up of T cells and dendritic cells, form one PP [8]. The domes of PPs are covered by a single layer of epithelial cells, the follicle-associated epithelium (FAE), consisting of enterocyte-like cells and membranous/microfold cells, so named because of their membranous appearance in electron micrographs [9], [10]. The M cell lacks an extensive glycocalyx, leading to greater exposure of the apical plasma membrane to the lumen of the GI tract, making this cell type specialized in sampling the gut lumen. The M cell has also fewer lysosomes and lower expression of digestive enzymes than surrounding enterocytes [11], while evidence suggests the expression of an antimicrobial peptide called peptidoglycan recognition protein S in the M cells [12]–[14]. A specific feature of M cells is transepithelial transport, more specifically the ability to endocytose soluble molecules as well as transcytose whole bacteria and other particulate matter from the lumen and deliver them to the immune cells in the subepithelial dome of the patch [11], [15], [16]. Uptake and transport of antigen and bacteria over the FAE is increased compared to regular villus epithelium, which results in higher amounts of transported material reaching the subepithelial dome [16]. Transepithelial transport of antigen and whole bacteria can either activate or inhibit the immune response, but is also an entry route for pathogenic agents such as bacteria, viruses, protozoa and prion particles [17]–[19]. The overall function of the PP in sampling gut luminal content and presenting antigens to the underlying immune cells in order to induce tolerance is well studied [20]. Much attention has been given to M cell morphology, function and differentiation [18], [19], [21]–[25]. But the study of M cells is complicated due to the lack of generally applicable specific markers for M cells. Therefore, they are commonly identified by staining with a combination of the lectin *Ulex europaeus* agglutinin-1 (UEA-1) and some other marker, such as antibodies directed against glycoprotein 2 (GP2) or NKM16-2-4, the double positive cells being classified as M cells. As a side effect, goblet cells are in these reports detected as UEA-1 positive but GP2 or NKM16-2-4 negative cells [18], [19], [21], [22], [24], [25]. There are examples of co-staining with UEA-1 and wheat germ agglutinin (WGA), the double positive cells being goblet cells, but also in these cases the focus is on M cell function [21], [23] and not goblet cell morphology or mucus properties. If goblet cells are quantified, they are used as a control for specific M cell depletion [18] and no specific goblet cell markers have been used in these reports [18], [19], [21]–[25]. Consequently, no consensus has been reached concerning the number of goblet cells on the domes and whether the PP is covered by a mucus layer [26]–[29]. We have now quantified not only mouse goblet cells in the FAE of ileal PPs, but also rat and human goblet cells in the corresponding tissue, using a specific antibody directed against the main component of intestinal mucus, the MUC2 mucin. In addition, we have studied properties of mucus covering PPs in ileal explants [30].

## Materials and Methods

### Animals and Ethics Statement

Male C57BL/6 mice (Taconic, Ry, Denmark) and male Wistar rats (B&K Universal AB, Sollentuna, Sweden) were housed in ventilated cages under pathogen free conditions with a 12 h light/dark cycle and access to standard chow and water *ad libitum*. Animals were anesthetized by isoflurane inhalation and killed by decapitation, all efforts being made to ease suffering. All mouse procedures were approved by the local Laboratory Animal Ethics Committee, University of Gothenburg, and rat procedures were approved by the ethical committee on animal experiments, Linköping University and were conducted in accordance with guidelines from the Swedish National Board for Laboratory Animals.

### Human Material

Macroscopically normal intestinal specimens of 5 patients were taken from the terminal ileum next to the ileocaecal valve during surgery for colonic cancer at Linköping University Hospital. The patients (1 male and 4 females, 67–92 years old) had macro- and microscopically normal mucosa and none had received preoperative chemo- or radiotherapy. The study was approved by the Regional Ethical Committee, Linköping, Sweden and all subjects gave their informed written consent.

### Mucus Thickness Measurements

Mucus measurements were performed as described previously. Briefly, the terminal ileum from mice was dissected, flushed with ice cold oxygenated (95% O_2_, 5% CO_2_) Krebs buffer (composition in mM: NaCl 116, CaCl_2_ 1.3, KCl 3.6, KH_2_PO_4_ 1.4, NaHCO_3_ 23, MgSO_4_ 1.2, pH 7.4). The ileum was opened along the mesenteric border and the longitudinal muscle layer removed by blunt dissection. From each mouse two large PPs were identified by eye and mounted in horizontal perfusion chambers, where the apical and basolateral solutions can be changed separately and in which the FAE was exposed to the serosal and mucosal solutions via 4.9 mm^2^ circular openings. After mounting, the apical part of the chamber was filled with 150 µl oxygenized room tempered Krebs buffer substituted with D-mannitol (10 mM), Na-pyruvate (5.7 mM), and Na-L-glutamate (5.1 mM), pH 7.4 (Krebs-mannitol). The basolateral side of the tissue was perfused with room tempered Krebs buffer containing D-glucose instead of D-mannitol (10 mM), pH 7.4 (Krebs-glucose), at a rate of 5 ml/h. Tissue temperature was controlled by placing the chamber in a heating block connected to a temperature controller (Harvard Apparatus, Holliston, MA) and the temperature gradually increased to 37°C during 10 min. The experiments were performed at this temperature. To avoid disturbing the mucus gel, the apical chamber was kept unstirred and at a constant volume. Tissue viability was monitored by measuring transepithelial potential difference (PD), using reference electrodes (Ref201, Radiometer, Copenhagen, Denmark) connected to the chamber via agar bridges (4% agar, 0.9% NaCl).

The transparent mucus layer was visualized through a stereomicroscope at 40× magnification (Leica MZ125, Wetzlar, Germany) by allowing activated charcoal particles in Krebs-mannitol to sediment onto the mucus surface. Images were acquired using a Leica IC D 3.3 megapixel camera mounted to the microscope, and mucus was aspirated with a Gilson Pipetman® P200 (Middleton, WI) set to 150 µl and a yellow tip (no. 70.760.502, Sarstedt, Nümbrecht, Germany). Mucus thickness was measured with micropipettes pulled from borosilicate capillaries with filament (OD: 1.2 mm, ID: 0.6 mm, Sutter Instruments, Novato, CA) to a tip diameter of 5–10 µm, using a P-97 Flaming/Brown Micropipette Puller (Sutter Instruments, Novato, CA). To attain the distance (D) between the charcoal particles and the epithelium, the micropipette was mounted in a micro-manipulator (in house) kept at a constant angle of 40°, connected to a digimatic indicator (Mitotoyo, Tokyo, Japan) The tip of the micropipette was adjusted to the same focal plane as the charcoal particles on top of the mucus and the position recorded. Then the focal plane was adjusted to the same focal plane as the epithelium and that position recorded. The distance between the two points represents D. The vertical thickness of the mucus was calculated by multiplying D with cos40 [31]. On villus epithelium mucus thickness measurements were made in two steps. First from charcoal particle to villus tip and then, after mucus was removed, villus height was measured from the epithelium between the villi to the villi tips. Total mucus thickness is presented as the sum of these two measurements. To obtain an average mucus thickness over the epithelial surface, five points were measured and the average thickness calculated. Initial mucus thickness was not measured on top of the domes, due to uncertainties as to whether mucus on top of the domes is released from the dome or from surrounding villi. Mucus release was stimulated by perfusing the explants basolaterally with a combination of the secretagogues carbachol and prostaglandin E_2_ (PGE_2_), 10 µM of each in Krebs-glucose for 40 min [32].

### Mucus Penetrability

Mouse ileum explants containing a PP were dissected and mounted in a horizontal imaging chamber (RC-50, Warner Instruments, Hamden, CT) with a 1.8 mm^2^ opening and 1.5 ml Krebs-mannitol buffer was added to the apical chamber. The serosal side was constantly perfused at 5 ml/h with Krebs-glucose solution containing CellTrace Calcein Violet, AM (1 µg/ml, Invitrogen, Carlsbad, CA) to visualize the tissue. During a period of 10 min the chamber was heated to 37°C, using a temperature controller (Harvard Apparatus, Holliston, MA) and thereafter kept at a constant temperature. After temperature equilibration, the apical buffer was removed and a suspension of 0.5 µm (red), 1 µm (far red) and 2 µm (green) fluorescent beads (FluoSpheres, Invitrogen, Carlsbad, CA) was added to the apical surface. The beads were allowed to settle in the mucus for 5 min before new Krebs-mannitol buffer was added to the apical chamber and the beads were left to sediment through the mucus for 30 min. Confocal Z-stacks (optical section 2.8 µm, interval 10 µm) were taken to evaluate the distribution of the different beads throughout the mucus, using an upright LSM 700 Axio Examiner 2.1 confocal imaging system with a Plan-Apochromat ×20/1.0DIC water objective (Carl Zeiss, Oberkochen, Germany). Volocity 5.5.1 (PerkinElmer, Waltham, MA) software was used to process images. Representative Z-stacks are shown to illustrate bead penetrability in mucus on top of mouse FAE.

### Histology and Transmission Electron Microscopy

Mouse, rat and human PPs were fixed in Karnovsky's fixative (2% paraformaldehyde, 2.5% glutaraldehyde in 0.05 M, sodium cacodylate buffer, pH 7.2) for 24 h followed by sequential staining using 1% OsO_4_ for 4 h, 1% tannic acid for 3 h and 1% Uranyl acetate overnight. Samples were dehydrated and embedded in epoxy resin (Agar 100, Agar Scientific, Stansted, UK). For light microscopy, 1 µm sections were cut, stained with Periodic acid-Schiff and images acquired with a Nikon eclipse 80i microscope, a Plan Apo 60× oil immersion objective, N.A. 1.40 and a Nikon digital camera (DXM 1200, Nikon Instruments Europe B.V., Amstelveen, The Netherlands). Electron microscopy was conducted on 50 nm sections cut using an Ultracut E (Reichert, New York, NY) microtome and collected on mesh copper support grids. The sections were contrasted using lead citrate and tannic acid and images were acquired using a Zeiss 902 electron microscope (Carl Zeiss, Oberkochen, Germany).

### Immunohistochemistry

Mouse, rat and human follicular tissue was fixed in Carnoy's fixative (60% dry methanol, 30% chloroform and 10% glacial acetic acid), embedded in paraffin and cut in 4 µm thick sections, which were dewaxed using Xylene substitute (Sigma, St. Louis, MO) and hydrated. Antigen retrieval was performed by microwave heating in 0.01 M citric buffer pH 6. The primary antibody used was custom made anti-MUC2C3 (1∶500) [1], detected with goat anti-rabbit Alexa 488 secondary antibody (Invitrogen, Carlsbad, CA) and DNA was stained by TO-PRO®-3 Iodide (1 µM, 642/661, Invitrogen, Carlsbad, CA) or DAPI (Invitrogen, Carlsbad, CA). Pictures were obtained by acquiring Z-stacks using an upright LSM 700 confocal imaging system (Carl Zeiss, Oberkochen, Germany). The goblet cells, defined as MUC2 positive cells and TO-PRO-3 positive epithelial cell nuclei, in FAE from 5 mice, 5 rats and 5 humans were counted by three independent researchers and the percentage of goblet cells to epithelial cells in the domes calculated for each section.

### Drugs

Carbachol and PGE_2_ (Sigma, St Louis, MO), were dissolved in water or a 1∶1 mixture of ethanol and DMSO at 10^−1^ M, respectively. Substances were then further diluted in Krebs-glucose buffer.

### Statistical Analysis

Data are presented as mean ± standard error of the mean (SEM) for n animals/subjects. The percentage of goblet cells in FAE is presented in a box graph as median with the 25^th^ and 75^th^ percentile and whiskers give the minimum and maximum values for n sections. Mann-Whitney test was used to test differences between two groups whereas for comparison between multiple groups, Kruskal-Wallis test followed by Dunn's post hoc test were employed. Statistical significance was accepted when *p*<0.05.

## Results

### Mouse explant Peyer's patches have a mucus layer

**Figure 1:**
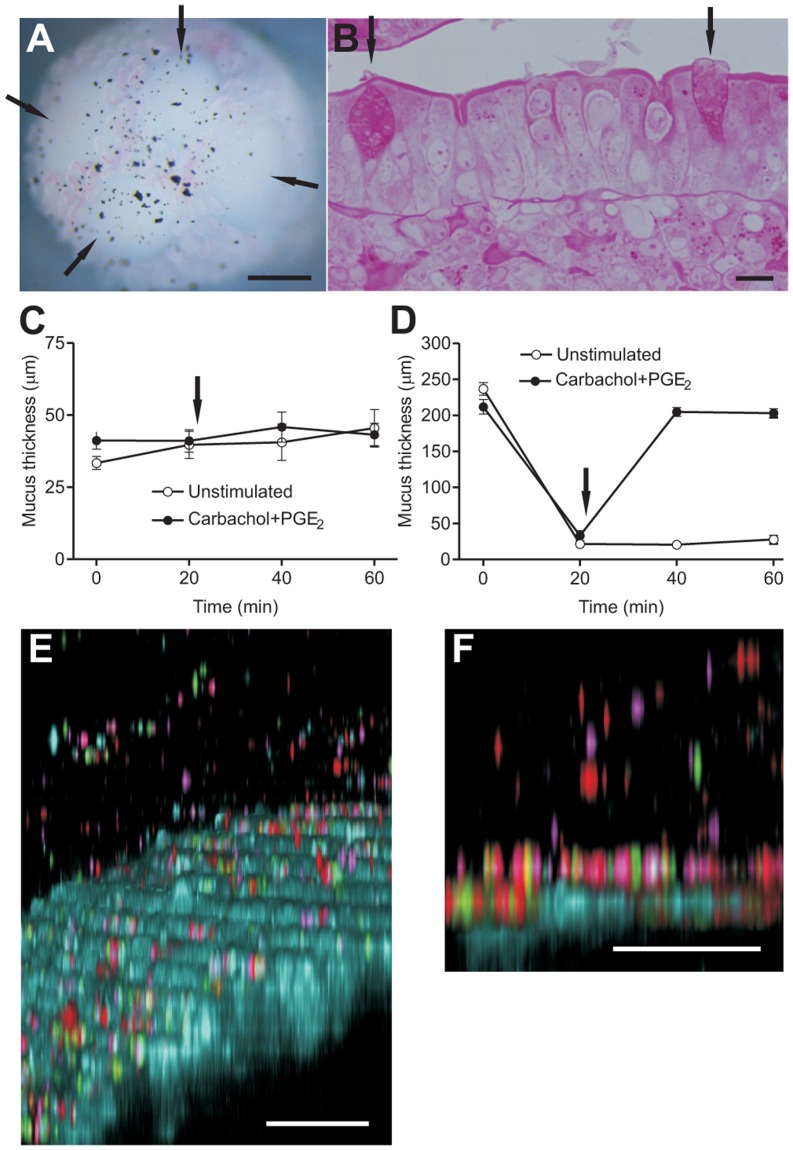
Mouse ileal Peyer's patches are covered by a mucus layer. (A) Stereo microscope image of an ileal explant containing a PP. Domes are indicated by black arrows. Charcoal particles were added to visualize the otherwise transparent mucus layer (bar = 0.5 mm). (B) Two mucus filled goblet cells (black arrows) in a dome stained by PAS (bar = 10 µm). (C) Mucus on top of the FAE was removed and remaining mucus thickness measured every 20 minutes for an hour (open circles; n = 6) or mucus thickness was measured at time 0 and 20 min and a combination of carbachol and PGE_2_, 10 µM of each, was perfused after the second measurement (arrow, closed circles; n = 6). (D) Initial mucus thickness was measured on the villi of the PP, mucus was removed and remaining mucus thickness measured at time 20 min. Half the number of explants were left unstimulated (open circles; n = 10) and half of the explants were stimulated with carbachol and PGE_2_ (10 µM of each; arrow), and mucus thickness was measured at time 40 and 60 min (closed circles; n = 10). (E) Mucus penetrability to beads the size of bacteria was assessed by confocal imaging of mouse ileal explants containing a PP. Tissue is visualized in blue and beads are red (0.5 µm), purple (1 µm) and green (2 µm). (F) To clarify how beads penetrate to the FAE surface, a flat section of the epithelium (blue) is shown. Note how some beads (red, purple and green) are suspended in the mucus. Bars in E and F = 50 µm.

In order to investigate mucus properties on PPs, explants from mouse ileum with PPs visible at dissection were mounted in the horizontal Ussing-type perfusion chamber and a suspension of charcoal particles in Krebs-mannitol was added to visualize the otherwise transparent mucus. The aggregated lymphoid follicles used for mucus measurements contained three or four domes with villi between them. Fig. 1A shows a brightfield image of four domes (Fig. 1A), acquired through a stereo microscope. Note that the charcoal particles are found on top of the mucus, well above the epithelium, indicating the presence of a mucus layer on top of the villi between the domes as well as on the actual domes. Several goblet cells situated in the FAE were detected in PAS stained epoxy resin imbedded sections from mouse ileal PPs (Fig. 1B).

We have previously shown how mucus release can be induced from ileal explants mounted in the perfusion chamber [30] using a combination of the secretagogues carbachol and PGE_2_ at 10 µM, but mucus release from PP goblet cells has not yet been studied, neither from the FAE nor the villi. Two explant tissue samples containing PPs from the same mouse were mounted in separate Ussing-type chambers and the original mucus layer was removed. Measurement of remaining mucus thickness on top of the domes was made at time 0 and 20 min. One PP was left unstimulated and the other was stimulated with 10 µM carbachol and 10 µM PGE_2_. Mucus thickness was measured at time 40 and 60 min in both cases and did not increase on top of the FAE, even after stimulation with carbachol and PGE_2_ (Fig. 1C). Another set of experiments was performed to study mucus growth on villi epithelium on mouse PPs (Fig. 1D). Initial mucus thickness was measured, mucus was removed and the remaining thickness measured. In one set of explants the tissue was left untreated and mucus thickness did not increase during the 40 min the tissue was left in the chamber. Upon stimulation with the same secretagogues as in C, however, mucus was released and a mucus layer could be measured on and between the villi (Fig. 1D).

An important property of GI tract mucus is penetrability to bacteria, which can be assessed by adding fluorescent beads the size of bacteria and allowing them to sediment in the mucus for a set amount of time. To investigate the penetrability to beads on top of PP domes, explants were mounted in the horizontal Ussing-type chamber and fluorescent beads with diameters of 0.5, 1 and 2 and µm were allowed to sediment into the mucus for 30 min, after which Z-stacks were acquired with a confocal microscope. The majority of the beads were found on the FAE, but some were suspended in the mucus (Fig. 1E), which is further evidence for the presence of a mucus layer on the domes of PPs.

### MUC2 immunopositive goblet cells are present in the follicle-associated epithelium

**Figure 2:**
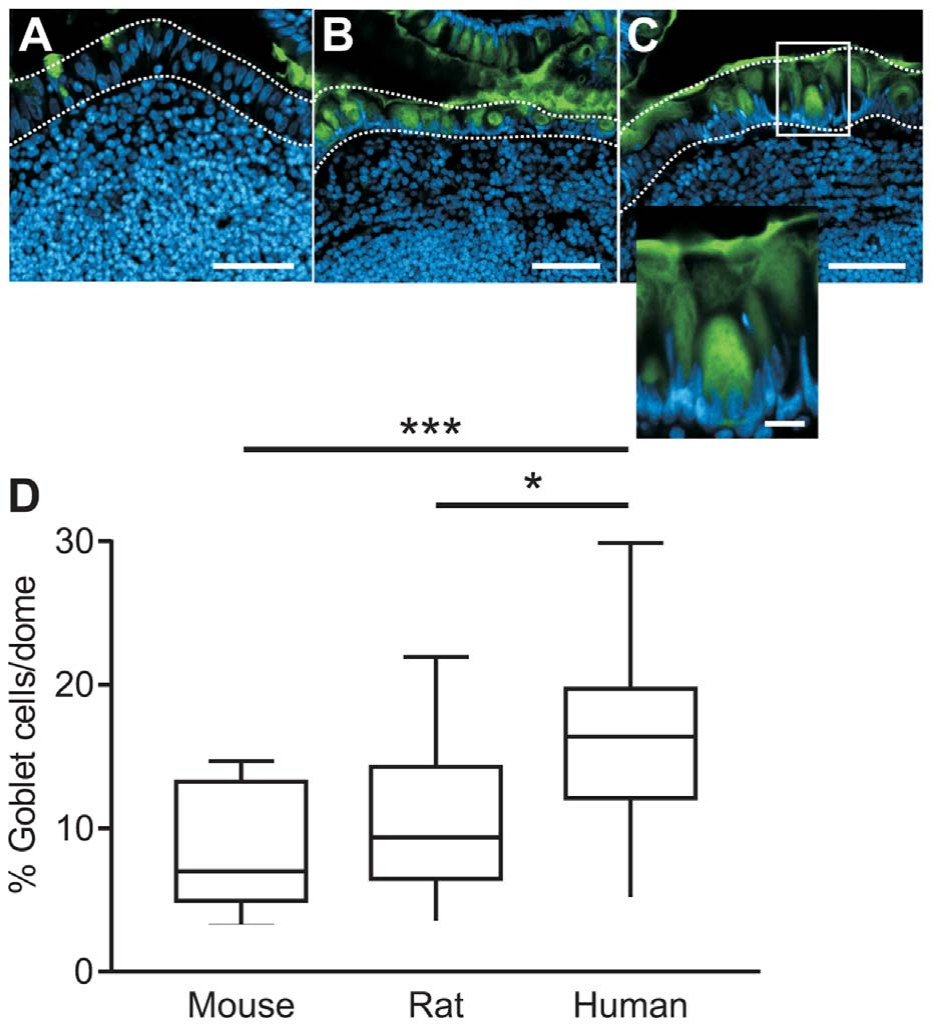
MUC2 positive cells on domes of mouse, rat and human Peyer's patches. Fluorescent staining of Muc2 reveals mucin containing cells in the FAE of a mouse (A), rat (B) and human (C) ileal PP. Bars = 50 µm. Inset in panel C shows the MUC2 positive cells at higher magnification (bar = 10 µm). Muc2 staining is green, nuclei are blue and FAE is indicated by dashed lines. (D) MUC2 positive cells and nuclei in FAE were counted in sections from 5 mice, 5 rats and 5 humans. Values are presented as median (25^th^ and 75^th^ percentile). The percentage of goblet cells was larger in human FAE compared to mouse FAE (*P*<0.001, ***) and rat domes (*P*<0.05, *).

As mucus was observed on top of the FAE and PAS positive cells were detected in sections of mouse domes, we asked if these cells were positive for MUC2 immunostaining, since MUC2 is the major gel forming mucin in the small intestine. Stainings with anti-MUC2C3 were performed on Carnoy fixed paraffin sections from ileal PPs (5 mice, 5 rats and 5 humans) and representative images of PP domes are shown for mice in Fig. 2A, rats in Fig. 2B and humans in Fig. 2C. To visualize the morphology of the FAE goblet cells, the epithelium within the white frame is shown in greater magnification (Inset Fig. 2C). Interestingly, FAE in all species examined contained MUC2 positive cells, thus identified as goblet cells. The median percentage of goblet cells to the total number of epithelial cells in FAE was 7.0 (25th percentile 5.0- 75th percentile 13) for mouse, 9.4 (6.5–14) for rat and 16 (12–20) for human sections (Fig. 2D). A higher percentage of cells was goblet cells in human PP domes compared to mouse domes (*P*<0.001, ***) as well as human domes compared to rat domes (*P*<0.05, *).

### Transmission electron microscopy reveals mucus releasing goblet cells on domes

**Figure 3:**
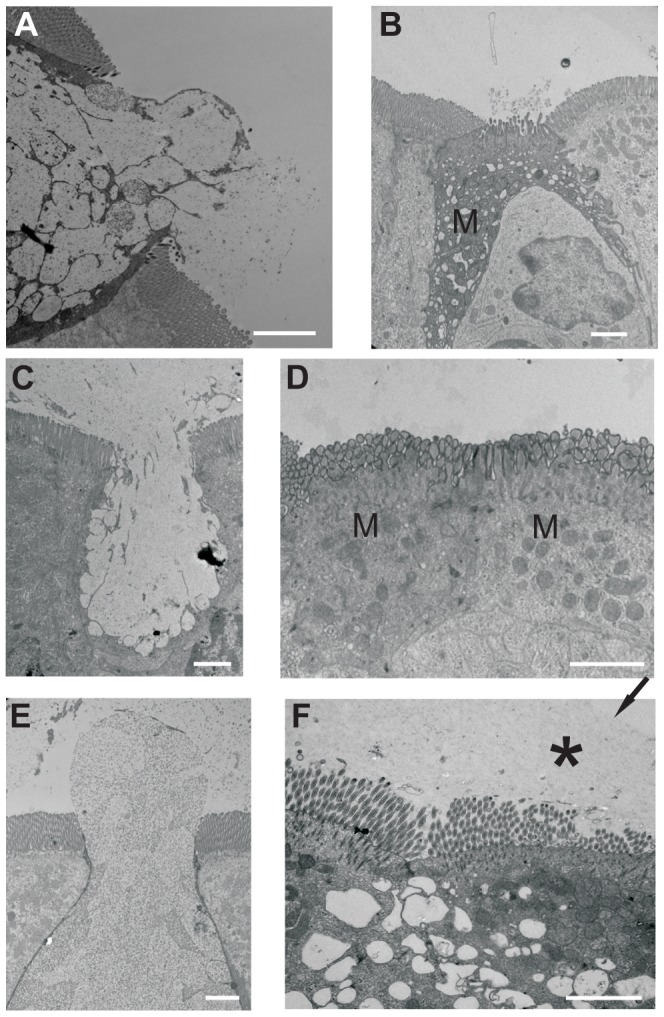
Transmission electron micrographs of mouse, rat and human Peyer's patches show secreting goblet cells. (A) Secreting goblet cell in mouse FAE. (B) M cell in mouse FAE. (C) Secreting goblet cell in a rat FAE. (D) Two M cells next to each other in a rat FAE. (E) Secreting goblet cell in human FAE. (F) Mucus on top of a human FAE, mucus border indicated by black arrow and mucus marked by black star. Bars = 2 µm.

Transmission electron microscopy on ultrathin sections of mouse, rat and human domes were used to visualize goblet cells and address if any of the goblet cells in the FAE were secreting mucus. In mouse FAE, a few secreting goblet cells were identified (Fig. 3A) as well as goblet cells with intact mucus granulae. In addition to goblet cells, we could also identify M cells in mouse FAE (Fig. 3B). Also in rat FAE, numerous secreting as well as intact goblet cells (Fig. 3C) and M cells (Fig. 3D) were found. Human FAE contained a substantial number of goblet cells, and some of them were secreting mucus (Fig. 3E). We could not find any cells on the human domes with a morphology resembling that described for M cells. In the human sections, a mucus layer covering the FAE was occasionally observed (Fig. 3F).

## Discussion

Reports on goblet cell number and mucus over the FAE of PPs are limited and contradicting, which led us to quantify goblet cells in FAE of mouse, rat and human PPs using a specific goblet cell marker, as well as investigating the properties of the mucus. It is generally agreed that PPs function as sites for surveillance of gut luminal content and induction sites for inflammatory immune responses as well as mucosal tolerance. The specialized epithelial cells called M cells, which reside in the FAE, possess a less dense glycocalyx compared to other enterocytes in addition to other features to facilitate uptake of soluble factors, particulate matter and whole microorganisms from the lumen and deliver them to the underlying immune cells. However, in addition to their function in inducing tolerance, the M cells in the FAE are also entry sites for pathogenic bacteria and viruses and the mucus layer could reduce the invasion of pathogens [7]. Mucus is a part of the mucosal barrier and the presence of a mucus layer on top of the PP could be considered to obstruct uptake of antigens via the FAE [1]. To address whether there is a mucus layer on top of PPs, we used the horizontal Ussing-type chamber to explore mucus release and expansion on top of PPs from the mouse ileum. Further, we calculated the percentage of goblet cells to epithelial cells in FAE from mice, rats and humans and also confirmed the presence of goblet cells and mucus on mouse, rat and human domes using transmission electron microscopy. We found that, as observed by others in rats [26], there is a mucus layer on top of the PPs of mouse ileum, both on the villi and on top of the FAE. We have recently shown that the mucus layer in the ileum is penetrable to fluorescent beads with sizes typical for bacteria [3]. Here we show that the easily removable mucus on top of the FAE is penetrable to the same type of beads.

M cell function and morphology have been extensively studied, for example due to their role in uptake of prion particles [18], [19], infection of type I pili-expressing pathogens [24] and targeting of mucosal vaccines [22]. However, the identification of M cells is confounded by the absence of specific markers for M cells. Therefore, in order to identify M cells, it is customary to combine staining with UEA-1 and for example antibodies directed against GP2 or the monoclonal antibody NKM16-2-4 [18], [19], [21]–[24], defining M cells as co-stained cells. Cells staining with UEA-1 but not with the GP2 or NKM16-2-4 antibodies are categorized as goblet cells and in these studies generally mentioned as a side effect of M cell identification. When studying prion uptake, mice were depleted of M cells, which was illustrated by a decrease in cells co-stained for UEA-1 and GP2. The number of cells positive for UEA-1 but negative for GP2 was not affected, thus pointing to a specific effect on M cells and no effect on goblet cells.

To our knowledge, there are only a few studies specifically focused on investigating the presence of goblet cells in the FAE, and results are contradicting. For example, Inamoto *et al* found that degenerated bacteria were occasionally engulfed by goblet cells in FAE of rat small intestine [33]. However, the number of goblet cells was not estimated. Similarly, Lelouard *et al* identified goblet cells in FAE of rabbit ileum and appendix [34]. Likewise, Onori *et al* found what they interpreted as a small number of goblet cells in rat ileal FAE [27]. They calculated the percentage of goblet cells in the FAE to be larger in the basal region (8.57±1.72%) compared to the apical region of the FAE (3.14±2.48%). Both these numbers are smaller than the number we found. In contrast, the recent publication by Beyaz *et al*, observed numerous goblet cells in isolated lymphoid follicles of the Angora rabbit jejunum, but no mucus-producing cells could be found on FAE of PPs [35]. These diverse reports suggest species differences regarding the presence and number of goblet cells present in the FAE of PPs and also variability depending on the location within the small intestine. The above mentioned studies mainly utilized electron microscopy to identify goblet cells by morphology. None has reported studies of mucus properties by measuring mucus thickness and penetrability in explants of PPs and none has quantified goblet cells from several PPs from each individual in three species using a marker specific for the MUC2 mucin. In the present study we identified a substantial number of goblet cells in ileal PP FAE from mice, rats and humans.

Recently goblet cells were indicated as antigen uptake points in the small intestine of mice and humans. Application of 10 kDa rhodamine Dextran as a model antigen to the intact mucosa revealed the formation of transepithelial dextran columns colocalizing with Muc2 staining. The columns were subsequently termed goblet cell-associated antigen passages (GAPs). The GAPs were distinct from M cell-like villous epithelial cells as they did not colocalize with GP2 staining. Additionally it was shown that the Dextran crossing the epithelium via these GAPs was taken up by CX3CR1^−^ CD103^+^ dendritic cells [36], which indicates a tolerogenic function [5], [7]. Even though the relative importance of these GAPs in relation to the FAE of PPs regarding the induction of tolerance is unknown, the phenomenon of GAPs indicates that goblet cells and mucus do not exclude uptake of antigens.

We detected no spontaneous growth of mucus from the patches, as in the rest of the small intestine. Upon stimulation with a combination of 10 µM carbachol and 10 µM PGE_2_, mucus was secreted from the villi on the PPs. In contrast, on top of the FAE no new mucus was formed after stimulation with the secretagogues for 40 min, despite positive stainings with an antibody directed against MUC2 and detection of mucus filled vesicles and mucus releasing cells in electron micrographs of domes. The combination of secretagogues used here induces mucus secretion in explants from the ileum, and other parts of the small intestine, whereas the distal colon is unresponsive [3]. Many of the physiological signaling molecules inducing mucus secretion *in vivo* are unknown, as is the physiological concentration of acetylcholine and/or PGE_2_ in the tissue. These observations suggest that mucus release from goblet cells in the FAE is controlled by other mechanisms than goblet cells in the rest of the small intestine.

It should be pointed out that the mucus found on top of the FAE is not anchored to the epithelium. This means that the mucus will be easily removed by intestinal peristalsis, exposing the FAE with its M cells to the luminal content. On the other hand, the mucus would be quickly replenished by mucus released from adjacent villi and again limit contact between the FAE and lumen.

To summarize, there is mucus on top of PPs in mice, rats as well as humans, a mucus layer penetrable to bacteria. However, the mucus on top of the FAE is not attached and peristalsis should remove it and bacteria contained in the mucus. The number of goblet cells relative to epithelial cells in human FAE is higher than in both mouse and rat FAE. Further, a direct coupling between goblet cells and dendritic cell sampling was recently shown [36], thus indicating that mucus secreting goblet cells do not exclude luminal sampling.
